# Energy Analysis and Heat Integration in the Joint Process of Biomass Fast Pyrolysis and In Line Sorption Enhanced Steam Reforming

**DOI:** 10.1021/acs.energyfuels.4c02555

**Published:** 2024-07-18

**Authors:** Pablo Comendador, Laura Santamaria, Maider Amutio, Jon Alvarez, Martin Olazar, Gartzen Lopez

**Affiliations:** † Department of Chemical Engineering, University of the Basque Country UPV/EHU, Barrio Sarriena s/n, Leioa, 48940, Spain; b Department of Chemical and Environmental Engineering, University of the Basque Country UPV/EHU, Nieves Cano 12, Vitoria-Gasteiz, 01006, Spain; c IKERBASQUE, Basque Foundation for Science, Plaza Euskadi 5, Bilbao, 48009, Spain

## Abstract

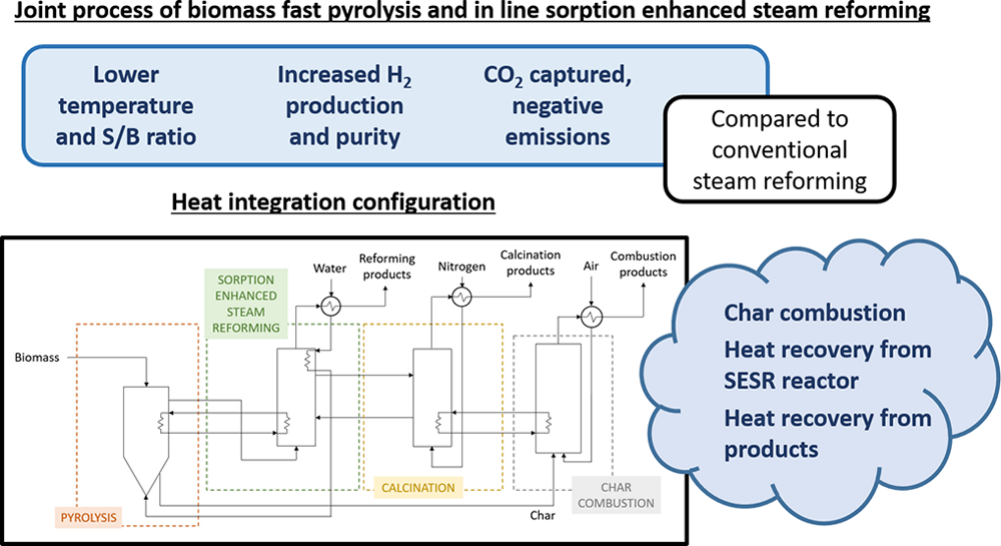

Biomass Fast Pyrolysis and in line Steam Reforming (PY-SR) is promising alternative for H_2_ production. However, there are potential strategies for intensifying the process, such as capturing the CO_2_ in situ in the reforming step, which is so-called Sorption Enhanced Steam Reforming (SESR). Both PY-SR and PY-SESR were simulated using a thermodynamic approach and empirical correlations, and they were compared based on the energy requirements, H_2_ production, and H_2_ purity at different temperatures (500–800 °C) and steam to biomass (S/B) ratios (0–4). Then, the energy requirements for the PY-SESR were analyzed in detail for a reforming temperature of 600 °C and several S/B ratios, and a heat integration scheme was proposed, aiming at making the process thermally autosustained. Although the energy requirement of PY-SESR is always higher than that of PY-SR at the same reforming conditions, it allows the use of milder operating conditions, with the process performance being even better. Thus, PY-SESR outshines PY-SR, as it allows obtaining a higher H_2_ production (0.124 kg_H_2__ kg^–1^_biomass_ vs 0.118 kg_H_2__ kg^–1^_biomass_) and H_2_ purity (98 mol % vs 67 mol %), with a lower energy requirement, and capturing the CO_2_ generated, thereby attaining negative emissions. The main energy demands of this process account for water evaporation and sorbent calcination. Nevertheless, a thermally autosustained PY-SESR process may be attained by recovering heat from the product streams, transferring heat from the reforming reactor to the pyrolysis reactor, and burning the char generated in the pyrolysis step.

## 1 Introduction

H_2_ is expected to play a key role in the energy mix in the coming years,^1,2^ with renewable sources, such as biomass, being a promising alternative to fossil ones for its production.^3^ Among the different methods of biomass valorization toward H_2_, gasification and bio-oil reforming are the most common ones,^4,5^ but the joint process of pyrolysis and in-line catalytic steam reforming is gaining attention, as it allows optimizing each step individually and avoiding the problems related to the condensation and volatilization of the bio-oil.^6−9^

However, the main problems to overcome are the fast deactivation of the reforming catalysts and the energy supply to the reforming step due to its endothermic nature.^10−12^ Besides, the product stream contains CO_2_, CO, and CH_4_, apart from H_2_,^13,14^ making it necessary to add further downstream purification stages, such as a Pressure Swing Adsorption (PSA) unit.^15,16^ This would allow obtaining a highly pure H_2_ product, but the off-gas containing CO_2_, CO, and CH_4_ would likely end up contributing to greenhouse gases emissions.

In this context, the use of a CO_2_ sorbent along with the catalyst in the reforming step may partially solve the problems mentioned above.^17,18^ The most common sorbents for this application are CaO-based ones, since they are low cost and compatible with the operating conditions of the steam reforming.^19,20^ Thus, the use of a CaO based sorbent would allow, on the one hand, CO_2_ removal from the gaseous media, promoting the displacement of the water shift reaction (WGS) and the steam reforming of methane and bio-oil oxygenates, thus obtaining an almost pure H_2_ product and higher conversions.^21^ On the other hand, the heat released due to the CO_2_ capture would attenuate the energy requirements of the endothermic steam reforming reactions.^22^

Although these benefits are promising, SESR requires a calcination step to regenerate the sorbent, which is an energy intensive operation.^23,24^ Different alternatives have been approached to deal with this calcination step, such as burning part of the feed in the calciner,^25^ or part of the H_2_ produced, using air or pure oxygen as oxidizing agents.^26^ There are also other process approaches, such as the chemical looping, where the use of an oxygen transfer material allows reducing the energy requirements.^27,28^ For example, Spragg et al.^29^ compared the bio-oil SESR with the bio-oil sorption enhanced chemical looping from a thermodynamic point of view, concluding that the latter involves a more favorable energy balance, but at the expense of a lower H_2_ production due to the oxidation of part of the feedstock.

Within this framework, this study aims to explore the PY-SESR process from an energy perspective. The authors have successfully developed an experimental configuration for the PY-SR of biomass, which is composed of a Conical Spouted Bed Reactor (CSBR) for the fast pyrolysis and a fluidized or fixed bed for the SR.^30−32^ Although this process was demonstrated to be successful for the valorization of different biomasses,^33^ its development toward a PY-SESR approach may be beneficial due to the reasons mentioned above. In short, it would allow overcoming the endothermicity of the reforming reactions with high conversion, producing a highly pure H_2_ stream and capturing the CO_2_. Nevertheless, the main challenge involved in this process lies in the energy required in the calcination of the sorbent, and therefore a heat integration strategy must be developed, which may also consider the combustion of the char (byproduct) generated in the pyrolysis step. Although char burning releases greenhouse gases when flue gases are not treated, the whole process would achieve negative emissions due to the use of biomass as a raw material and CO_2_ capture in the reforming step.

The approach selected for carrying out this study was based on thermodynamic calculations, empirical correlations, and previous experimental results. Thus, the heats of the reactions of biomass pyrolysis and char combustion were determined based on empirical correlations. The SR and SESR steps were modeled by using the Gibbs free energy minimization method, which has been proven a reliable alternative to reproduce experimental results in similar configurations as the one proposed in this study.^34−36^ The composition of the stream feed into the reforming step (biomass pyrolysis volatiles) was set based on previous experimental studies carried out in our facilities.^37^ The other operations were modeled through simple thermodynamic calculations for heat transfer and chemical reactions.

## 2 Materials and Methods

### 2.1 Process Description

As aforementioned, the PY-SR process has been studied in detail in previous experimental studies in our facilities.^13,30,32^ Thus, the process consists of feeding the biomass into a CSBR, using steam as the fluidizing agent. The biomass pyrolysis volatiles formed along with the steam are then fed into the reforming reactor, which may be either a fixed or fluidized bed. When reforming is conducting on Ni-based catalysts, a product stream composed of mainly H_2_, CO, and CO_2_ is obtained.

Studies have also been reported in the literature, in which the sorption enhanced steam reforming of bio-oil^25,38^ has been addressed. In this process, a sorbent is mixed with the catalyst to capture the CO_2_ generated in situ. However, in this approach, once the sorbent is saturated and the catalyst is deactivated, there is a need for regenerating the sorbent for subsequent use in cyclic operations. Studies dealing with this strategy have also been reported.^39^ At the lab scale, the regeneration process is usually carried out in the same reactor after the reaction step. However, industrial implementation of this process requires another reactor for the continuous regeneration, which consists of calcining the materials. There are examples of this regeneration reactor (calciner), in which methane SESR is conducted^40^ and chemical looping technology is used.^41^

**Figure 1:**
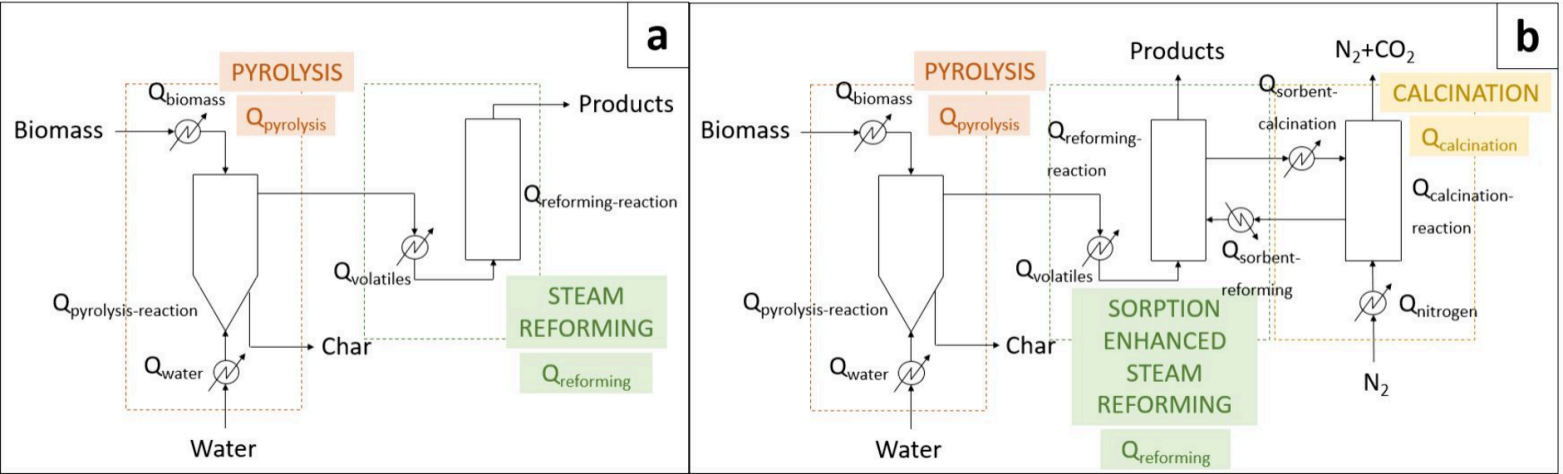
Flow diagrams showing the locations in which energy is required (*Q*). a: PY-SR; b: PY-SESR.

Concerning the PY-SESR process, there are hardly any studies reported in the literature, and the main differences with the PY-SR process lie in the use of a mixture of sorbent and catalyst in the reforming step and the need for another reactor to carry out the regeneration when operating in continuous mode. The process flow diagrams of both PY-SR and PY-SESR options are shown in Figure 1. It is worth noting that the heat exchanger symbols were only used to highlight the energy requirements.

The overall energy balance of the PY-SR configuration, in terms of *Q* (MJ h^–1^), is as follows:
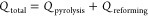
1The overall energy balance of the PY-SESR configuration, in terms of *Q* (MJ h^–1^), is as follows:
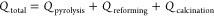
2where *Q*_total_ is the total energy requirement (MJ h^–1^), *Q*_pyrolysis_ is the energy requirement associated with the pyrolysis step (MJ h^–1^), *Q*_reforming_ is the energy requirement associated with the reforming step (MJ h^–1^), and *Q*_calcination_ is the energy requirement associated with the calcination step (MJ h^–1^).

It is worth mentioning that the terms in the previous equations are global ones, and they include all the energy requirements corresponding to the processes shown in Figure 1. Thus, *Q*_pyrolysis_ was determined as follows:
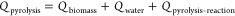
3where *Q*_biomass_ is the energy requirement for heating the biomass from ambient temperature to the pyrolysis one (MJ h^–1^), *Q*_water_ is the energy requirement for heating the water from ambient temperature to the pyrolysis one (MJ h^–1^), and *Q*_pyrolysis-reaction_ is the energy requirement for the pyrolysis reaction (MJ h^–1^).

Furthermore, H_2_ production was defined as follows:
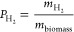
4where *P*_H_2__ is the H_2_ production (kg_H_2__ kg^–1^_biomass_), *m*_H_2__ is the mass flow rate of H_2_ produced (kg_H_2__ h^–1^), and *m*_biomass_ is the biomass mass flow rate fed into the pyrolysis step (kg_biomass_ h^–1^).

The PY-SESR process was then further analyzed, aiming at making it thermally autosustained. Accordingly, the char generated in the pyrolysis step was proposed to be used as a fuel in a combustor coupled with the calciner. Char from wood pyrolysis was proven to be suitable for power applications,^42^ and therefore the combustor may supply the heat needed for the calciner.^43^

**Figure 2:**
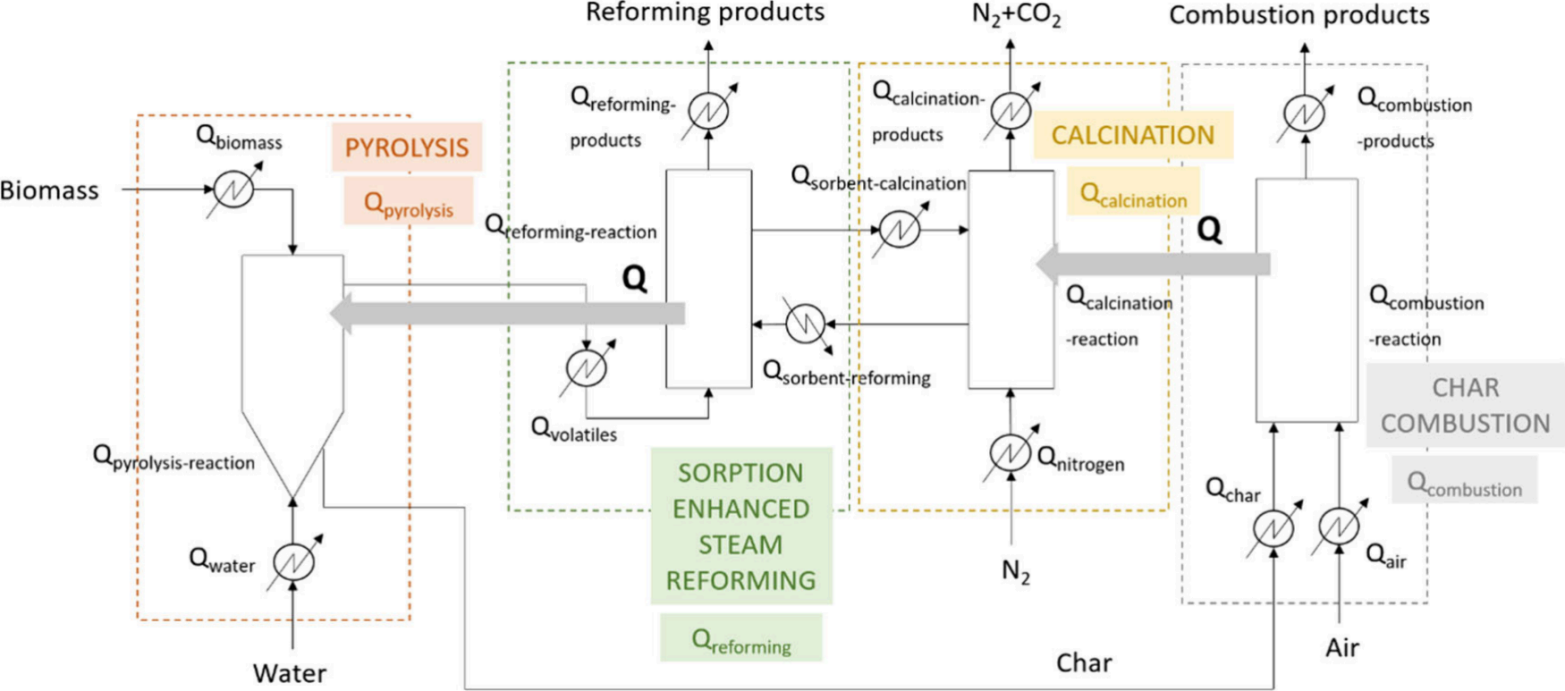
PY-SESR process flow diagram showing the locations in which energy is required for heat integration.

**Figure 3:**
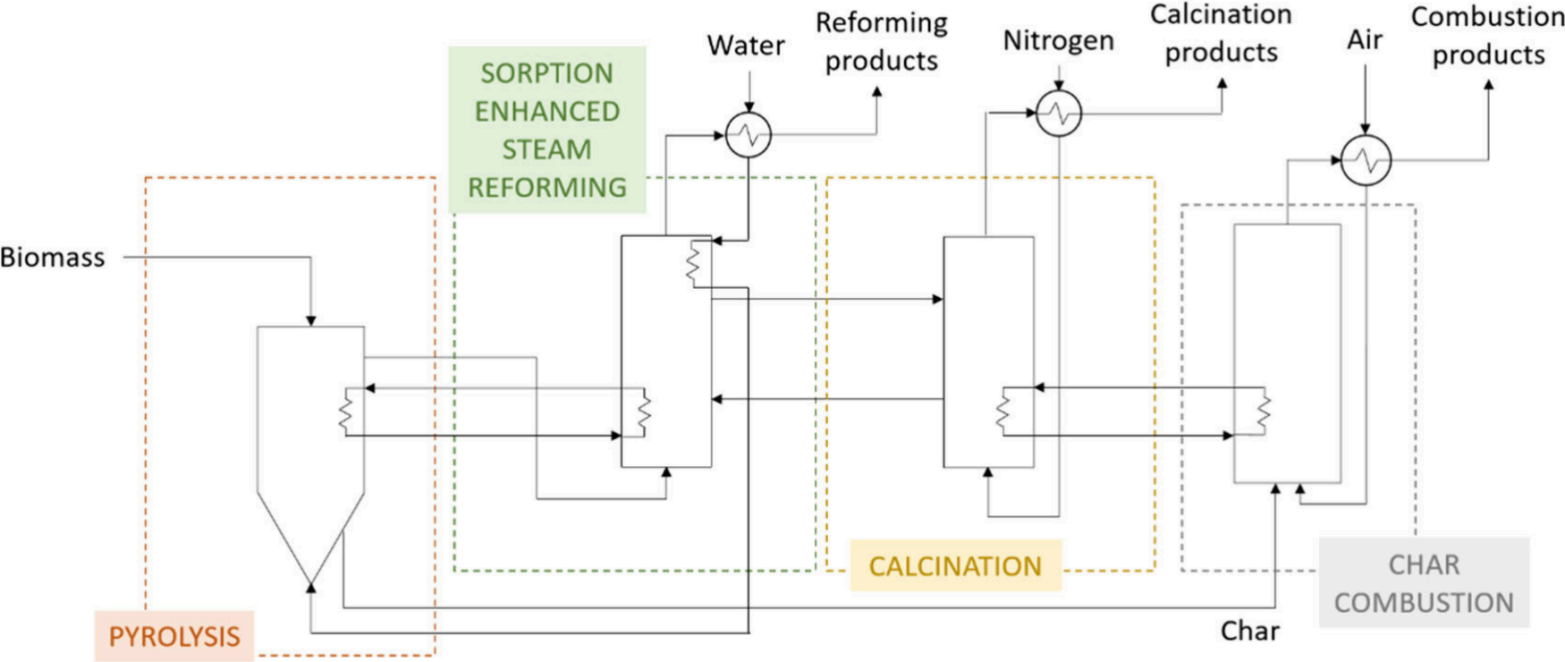
PY-SESR heat integration configuration.

The flow diagram showing the locations in which energy is required for PY-SESR to be thermally autosustained is shown in Figure 2. The proposed heat integration configuration is shown in Figure 3.

The overall energy balance may be summarized as follows:
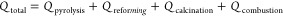
5where *Q*_combustion_ is the energy requirement associated with the combustion step (MJ h^–1^).

The configuration proposed for heat integration is based on the following considerations: (i) heat is supplied to the calciner by coupling it with a combustor in which the biochar generated in the pyrolysis unit is used as fuel, (ii) heat is recovered from the SESR reactor and supplied to both the pyrolysis reactor and the water feed to make the pyrolysis step energy balanced, and (iii) heat is recovered from the products streams. The idea is to make use of these streams to attain a flexible process concerning energy. In particular, the reformed product stream containing the unreacted water will be used for heating the water fed into the pyrolysis reactor; the product stream leaving the calciner will be used for heating the nitrogen feed, and the combustion products will be used for heating the air feed.

A detailed overview of the calculation procedure can be found in the Supporting Information.

### 2.2 Methodology and Data

#### 2.2.1 Reaction Steps

The pyrolysis temperature was set at 500 °C, and the value assumed for the pyrolysis heat of reaction was −0.255 MJ kg^–1^_biomass_.^44^ The temperature was selected based on previous experimental results,^37^ in which this value was found to be the optimum one for maximizing the bio-oil yield from pine wood sawdust. Although recent studies^8^ suggest that a higher pyrolysis temperature (up to 800 °C) may attenuate catalyst deactivation in the reforming step, it would involve a higher energy requirement, so 500 °C was assumed as the best choice in this study.

**Figure 4:**
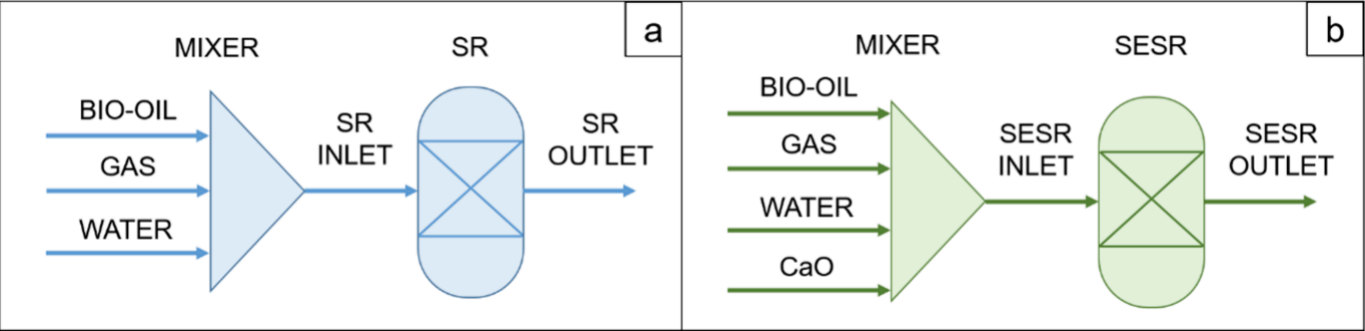
Pro II software process flow diagrams. a: SR option; b: SESR option.

Both SR and SESR were studied at operation temperatures between 500 and 800 °C. According to the experimental results, a temperature of 600 °C or higher is required for achieving high reforming reaction rates, and therefore full conversion in the SR of biomass pyrolysis volatiles.^30^ As for the SESR of bio-oil model compounds, temperatures of around 575 °C are the optimum ones.^45^ Besides, the temperature range in which CaO is active for CO_2_ capture is between 500 and 700 °C.^19,46^ In particular, the reforming reaction step was modeled using Pro II v.2021 software by means of an isothermal Gibbs reactor operating at constant pressure (1 atm) and under the Soave–Redlich–Kwong equation of state. The H_2_ production (kg_H_2__ kg^–1^_biomass_) and H_2_ purity of the reforming product stream (mol %) were used as the main reaction indices. The Pro II process flow diagrams are shown in Figure 4.

The composition of the inlet volatile stream was set according to previous studies of biomass fast pyrolysis performed in a conical spouted bed reactor at 500 °C.^34^ The pyrolysis fractions obtained and their yields when subjecting the biomass (pine wood sawdust) to fast pyrolysis under the conditions mentioned are given in Table 1. The biomass composition has been reported elsewhere.^30^

**Table 1:** Pine Wood Sawdust Pyrolysis Fractions and Their Yields^37^

fraction	yield (wt %)
Bio-oil	75.33
Gas	7.33
Char	17.34

The compositions of the bio-oil and the noncondensable gaseous fraction are shown in Table 2 and Table 3, respectively. Note that the main compound families in the bio-oil are set out in Table 2, but more specific information can be found elsewhere.^37^

**Table 2:** Composition of the Bio-Oil from Pine Wood Sawdust Pyrolysis at 500 °C^37^

compound	concentration (wt %)
Water	33.67
Phenols	21.89
Ketones	8.46
Saccharides	5.92
Furans	4.41
Acids	3.62
Alcohols	2.65
Aldehydes	2.56
Unidentified	16.82

**Table 3:** Composition of the Gaseous Fraction from Pine Wood Sawdust Pyrolysis at 500 °C^37^

compound	concentration (wt %)
Carbon monoxide	45.47
Carbon dioxide	45.33
Methane	4.99
Ethylene	1.25
Propylene	0.97
Ethane	0.83
Propane	0.69
2-Butene	0.42
Hydrogen	0.06

Based on the previous information, the average molecular formula of the pine wood sawdust pyrolysis volatile stream (excluding water) is CH_1,26_O_0,39_. Therefore, the overall chemical reaction for the steam reforming of this stream is as follows:
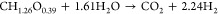
6Note that this simulation approach using an isothermal Gibbs reactor has been used successfully in several studies by our group.^34−36^ Thus, this simulation tool is especially accurate when experimental results are obtained under full conversion conditions (high catalyst space times and suitable temperatures and S/B ratios), i.e., close to thermodynamic equilibrium conditions.

The calcination temperature has a great influence on decarbonation kinetics and equilibrium CO_2_ partial pressure.^47,48^ For example, at 700 °C, the equilibrium CO_2_ partial pressure is 0.030 bar, so a diluting gas, such as N_2_, will be required for carrying out decarbonation. Furthermore, decarbonation kinetics are slow under these conditions. However, the materials (sorbent and catalyst) suffer less from sintering. At a higher temperature, such as 950 °C, the equilibrium CO_2_ partial pressure is 2.22 bar, so it would be possible to carry out the decarbonation with pure CO_2_ at atmospheric pressure, and the kinetics is fast. Nevertheless, the materials used must be resistant to sintering. In particular, and aiming at reaching a compromise, the calcination temperature was maintained in the 750–800 °C range.

The energy requirement in the calcination process was determined based on the heat of the reaction of calcium carbonate decomposition. Given the standard reaction enthalpy is 0.178 MJ mol^–1^,^49^ the value corresponding to the actual calcination temperature was calculated.

The combustion was carried out at 20 °C above the calcination temperature in order to ensure a thermal gradient for heat transfer from the combustion step to the calcination one. The energy related to the combustion of the char was determined based on its heating value, which is 30.4 MJ kg^–1^_char_.^37^

All steps were considered to operate at atmospheric pressure.

More information related to the calculations performed in each step can be found in the Supporting Information.

#### 2.2.2 Streams

The biomass considered was pine wood sawdust, since it is an abundant biomass waste in Europe, and its pyrolysis was studied as reported in previous papers in our facilities.^30,37^ The biomass mass flow rate was set at 100 kg h^–1^.

The water feed rate depends on the Steam to Biomass (S/B) ratio, which was varied between 0 and 4 kg_water_ kg^–1^_biomass_. The stoichiometric value of the S/B ratio required to reform the pyrolysis volatiles to the full extent is 0.55 kg_water_ kg^–1^_biomass_. However, as proven in a previous study,^30^ a higher value (up to 5) improves H_2_ production in the PY-SR process, although at the expense of a higher energy requirement. In addition, the use of S/B ratios above the stoichiometric one is critical to ensure high steam partial pressure in the reforming reactor and attenuate coke deposition on the catalysts, and therefore catalyst deactivation.^50−52^ Furthermore, S/B ratios are lower in PY-SESR than in PY-SR, as the water gas shift reaction is displaced by capturing CO_2_ in situ. Therefore, we considered that the range between 0 and 4 kg_water_ kg^–1^_biomass_ is that of interest for comparing the two processes.

Based on previous studies,^37^ the char yield in the pyrolysis step was set at 0.1734 kg_char_ kg^–1^_biomass_. Its detailed composition has also been reported elsewhere.^37^ In the PY-SESR process, the calcium oxide mass flow rate considered in the feed into the reforming reactor was 1.59 kg_CaO_ kg^–1^_biomass_, which corresponds to the stoichiometric amount of calcium oxide needed to capture all the CO_2_ formed if the biomass volatiles were fully reformed.

The nitrogen mass flow rate to feed into the calcination step was determined in order to reach a CO_2_ partial pressure 30% lower than that of equilibrium, which depends on the calcination temperature. The air mass flow rate supplied to the char combustion step was that required according to the stoichiometry for burning the char completely. The temperature of the inlet streams (biomass, water, nitrogen, and air) was set at 25 °C.

When the aim was to compare PY-SR and PY-SESR options, the temperatures of the outlet streams (char, reforming products, calcination products, and combustion products) were those of the steps where they are formed. However, in the SESR option with heat integration, a certain amount of heat was recovered in each product streams. Therefore, the outlet temperatures depend on the heat removed from the streams.

The specific heats of the biomass and the char were taken from the literature,^44^ as well as the remaining specific heats.^49^ Note that heat exchange rates between streams involving multiple compounds, such as those at the outlet of the pyrolysis or the reforming steps, were modeled by Pro II v.2021 software. More information regarding the heat exchange calculations involving the streams can be found in the Supporting Information.

## 3 Results and Discussion

### 3.1 Comparison of PY-SR and PY-SESR Processes

The PY-SR and the PY-SESR conventional configurations (Figure 1) were compared in terms of the total energy requirement, H_2_ purity, and H_2_ production. Different reforming temperatures and S/B ratios were studied. The pyrolysis temperature was set at 500 °C in both cases, and the calcination temperature in the PY-SESR option was set at 800 °C.

It is worth mentioning that the ideal working conditions (reforming temperature and S/B ratio) would be those allowing the lowest *Q* with the highest H_2_ purity.

**Figure 5:**
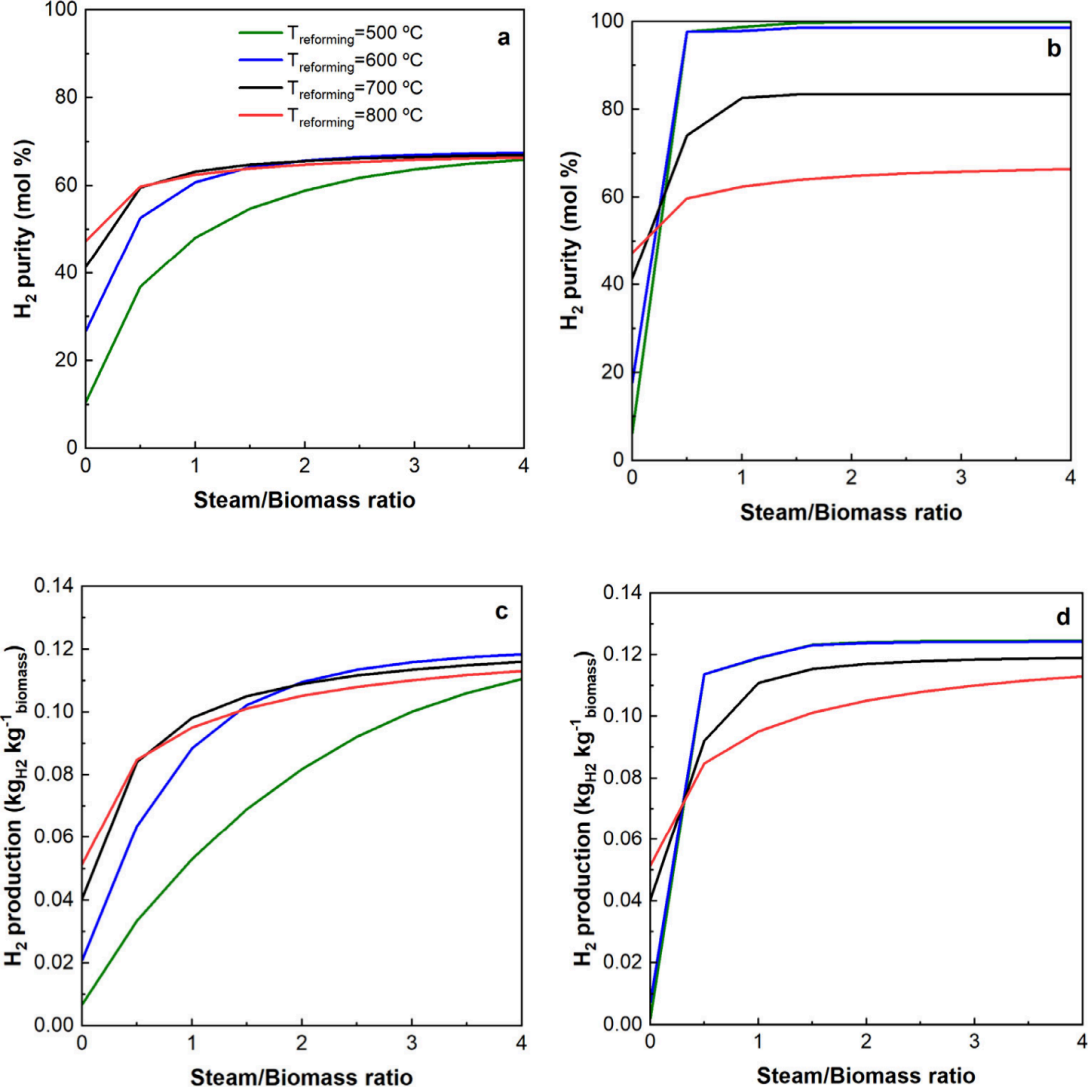
Effect of reforming temperature and S/B ratio on H_2_ purity and H_2_ production. a, c: PY-SR; b, d: PY-SESR.

The results corresponding to the H_2_ purity and H_2_ production are shown in Figure 5.

It can be observed that PY-SESR allows one to obtain higher H_2_ productions, and especially higher H_2_ purities, when operating at low temperatures and S/B ratios. Thus, using an S/B ratio of 1 and 500 °C, H_2_ production and H_2_ purity were 0.05 kg_H_2__ kg^–1^_biomass_ and 48.16 mol % for PY-SR, and 0.12 kg_H_2__ kg^–1^_biomass_ and 98.78 mol % for PY-SESR. These trends have already been reported in the literature for the sorption enhanced steam reforming of bio-oil.^25,53^

These results are explained by the removal of CO_2_ from the reaction environment, which causes the displacement of the water gas shift reaction in the presence of a metal catalyst. Furthermore, steam reforming of CH_4_ and oxygenates is enhanced by CO depletion.^54^ Thus, if the sorbent is active and there is enough steam in the reaction environment, all of the biomass pyrolysis volatiles are converted into H_2_ and CO_2_, with the latter being captured.

The sudden changes observed around an S/B ratio of 0.5, mainly in PY-SESR, are related to the fact that the stoichiometric value of the S/B ratio needed to reform all of the biomass volatiles is 0.55 kg_water_ kg^–1^_biomass_. In particular, it accounts for the pyrolysis volatile production from biomass (0.8266 kg_volatiles_ kg^–1^_biomass_) and for the water needed for converting those pyrolysis volatiles completely into H_2_ and CO_2_, according to stoichiometry. Below this value, there is no enough water for the full extent of the reforming reactions.

Furthermore, it can also be noticed that the temperature has a great influence on the PY-SESR performance. This is due to the promotion of calcium carbonate decomposition at high temperatures. In fact, the sorbent is not yet active at 800 °C, and therefore the performance of PY-SESR is very similar to that of PY-SR. This is consistent with the studies reported in the literature about the temperature window for use of calcium oxide to capture CO_2_ at moderately low CO_2_ partial pressures.^55,56^

Concerning the results for H_2_ production and purity, Arregi et al.^6^ reviewed in detail the thermochemical processes for H_2_ production from biomass. The following studies were mentioned. Niu et al.^57^ carried out steam gasification of pine wood sawdust, obtaining a H_2_ production of 0.062 kg_H_2__ kg^–1^_biomass_ and a H_2_ purity of 35 mol % at a temperature of 850 °C. Another strategy for H_2_ production is the steam reforming of raw bio-oil. Thus, Seyedeyn-Azad et al.^58^ obtained 0.123 kg_H_2__ kg^–1^_bio-oil_ with a H_2_ purity of 65.3 mol % at 950 °C and using a steam to carbon molar ratio of 5. As for the pyrolysis and in line steam reforming, Arregi et al.^30^ obtained 0.117 kg_H_2__ kg^–1^_biomass_ with a purity of 66 mol % at a temperature of 600 °C and using an S/B ratio of 4. It should be emphasized that although this last option (PY-SR) is very promising, there is room for further improvements, as proven in this study. In particular, the simulation results obtained for the PY-SR option predict a H_2_ production and purity of around 0.118 kg_H_2__ kg^–1^_biomass_ and 67 mol %, respectively, which were quite similar to those obtained experimentally by Arregi et al.^30^ However, according to the predictions for the PY-SESR option, a H_2_ production of 0.124 kg_H_2__ kg^–1^_biomass_ and a H_2_ purity of 99.9 mol % could be obtained.

**Figure 6:**
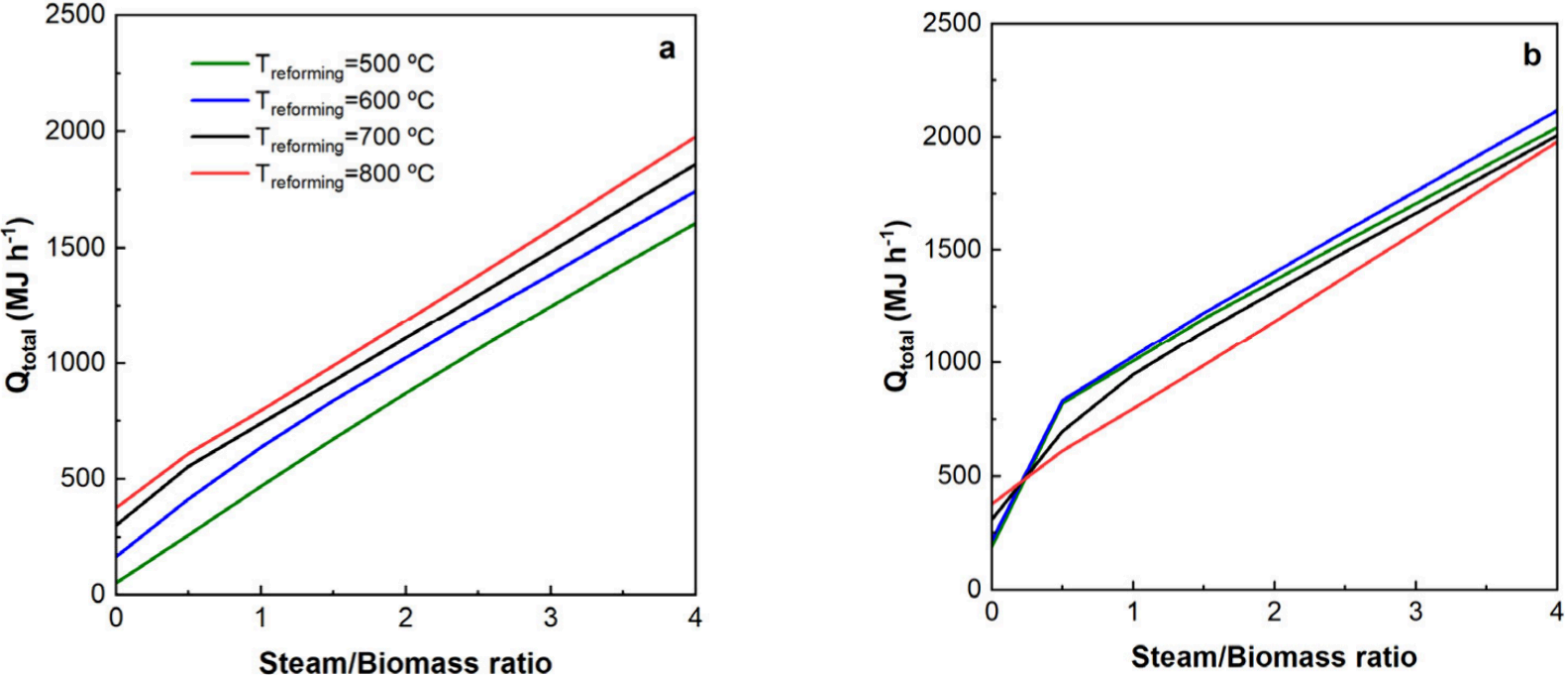
Effect of the reforming temperature and S/B ratio on *Q*_total_. a, PY-SR; b: PY-SESR.

Figure 6 shows the effect of the reforming temperature and S/B ratio on the overall heat requirement in both options.

In the PY-SR option, *Q*_total_ increases as the S/B ratio is increased independently of the reforming temperature, which is attributed to the increase in the heat requirements for steam generation. Furthermore, it can be observed that *Q*_total_ increases as the reforming temperature is increased for all S/B ratios, which is related to the fact that the gap between the pyrolysis temperature (500 °C) and the reforming temperature is higher, so the energy requirement for heating the pyrolysis volatiles is higher.

In the PY-SESR option, *Q*_total_ also increases with the S/B ratio independently of the reforming temperature for the same reason mentioned above. As for the effect of the reforming temperature, it is different depending on the S/B ratio. When an S/B ratio higher than 0.5 is used, *Q*_total_ decreases when the temperature is above 600 °C, which is due to the lower sorbent efficiency caused by the shift in the equilibrium of the carbonation reaction (the calcination energy requirements are lower). At 500 °C, *Q*_total_ is slightly lower compared to that at 600 °C due to the lower energy requirements for heating the pyrolysis volatiles. When the S/B ratio is lower than 0.5, the effect of the reforming temperature is reversed because there is not enough water for the full extension of the reforming reactions, so there is not enough CO_2_ for the full extension of the exothermic carbonation reaction.

In short, in the PY-SR option, a minimum temperature of 600 °C and an S/B ratio greater than 1 would be desirable for attaining a good performance concerning H_2_ production and purity. As observed in Figure 6a, the lower both the S/B ratio and the reforming temperature, the lower the energy requirements. In the PY-SESR option, a maximum temperature of 600 °C and a minimum S/B ratio of 0.5 allow the best results to be obtained in terms of H_2_ production and purity. As far as the heat requirements are concerned, the values are quite similar at reforming temperatures equal to or lower than 600 °C. Furthermore, as the S/B ratio is lower, *Q*_total_ is also lower.

Although the previous analysis is useful for a preliminary comparison of the two processes, it is important to mention that the S/B ratio and the reforming temperature have more implications than their effect on the energy requirements or equilibrium conversion. Thus, S/B affects also the fluidization regime of the pyrolysis step, the reforming reaction rate, and the catalyst deactivation.^50^ The reforming temperature highly affects the kinetics of the reforming reactions, which are hindered at temperatures lower than 550 °C.^30^

**Figure 7:**
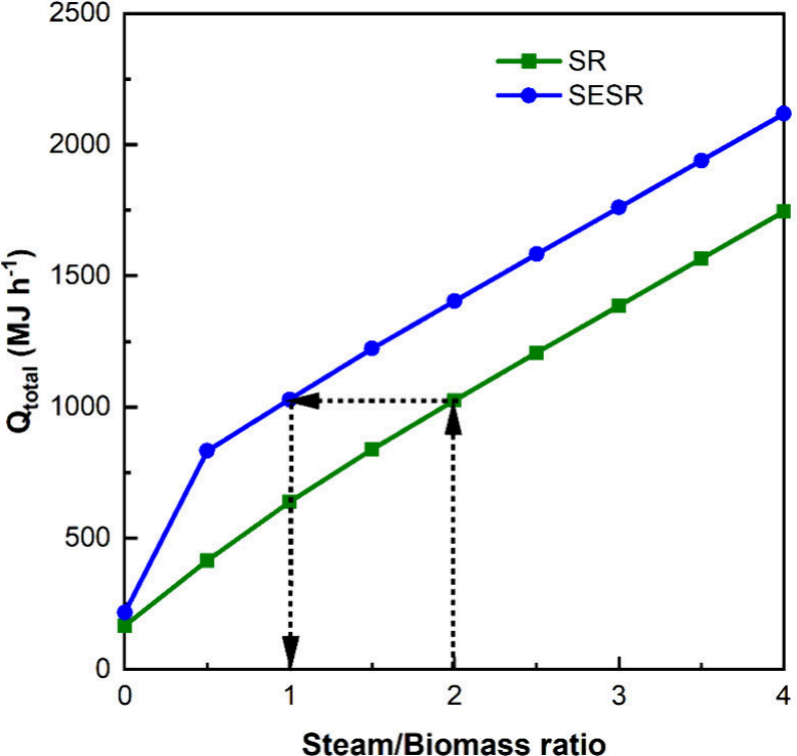
Influence of the S/B ratio on the energy requirements in PY-SR and PY-SESR options at a reforming temperature of 600 °C.

As a final remark, the values of *Q*_total_ are always higher for PY-SESR than for PY-SR when the operation is conducted at the same conditions of the reforming temperature and S/B ratio. Nevertheless, PY-SESR allows using milder operating conditions than PY-SR, with H_2_ production and purity being even higher, and therefore, operation with lower energy requirements may be conducted. This concept is illustrated in Figure 7 for a reforming temperature of 600 °C.

Figure 7 allowed determination of the equivalence of S/B ratios in PY-SR and PY-SESR options to achieve the same *Q*_total_ in the S/B_PY-SR_ ratio range of 1.5–4:
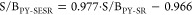
7Thus, using an S/B ratio of 2 in the PY-SR option, H_2_ production is 0.109 kg_H_2__ kg^–1^_biomass_, H_2_ purity is 65.71 mol %, and *Q*_total_ is 1025.07 MJ h^–1^. The same *Q*_total_ would be achieved in the PY-SESR option when using an S/B ratio of 1, with H_2_ production and H_2_ purity being 0.119 kg_H_2__ kg^–1^_biomass_ and 97.82 mol %, respectively.

### 3.2 Heat Integration in PY-SESR

#### 3.2.1 Overview of PY-SESR Heat Requirements

**Figure 8:**
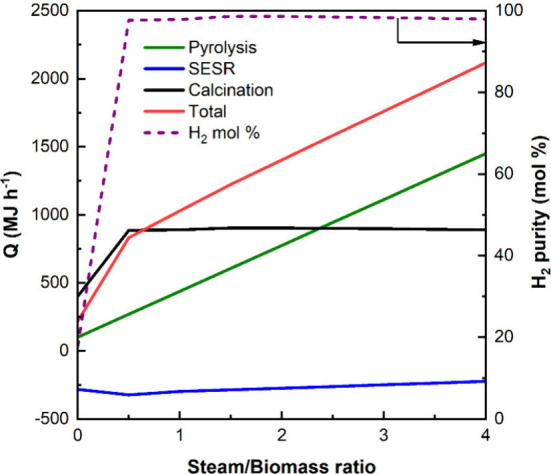
Influence of the S/B ratio on *Q* and H_2_ purity in the different steps of PY-SESR at a reforming temperature of 600 °C.

The PY-SESR option has been further studied by splitting the *Q*_PY-SESR_ into the *Q* required for each one of the steps, which are pyrolysis, SESR, and calcination. Figure 8 shows the dependency of *Q* and H_2_ purity with an S/B ratio at a reforming temperature of 600 °C. According to experimental results of both PY-SR of biomass^30^ and SESR of bio-oil model compounds,^45^ this temperature should be enough to avoid kinetic limitations under real process conditions. Besides, temperatures well above that lead to a decrease in H_2_ production and purity due to the sorbent loss of efficiency.

It can be observed that for all of the S/B ratios studied, the pyrolysis and the calcination steps require energy, whereas SESR provides energy. H_2_ purity and *Q*_calcination_ are stable when an S/B ratio above 0.5 is used. Below an S/B ratio of 0.5, *Q*_calcination_ decreases due to the lower CaCO_3_ formation due to the insufficient water amount for reforming all of the feed. As observed, *Q*_reforming_ is rather stable within the S/B ratio window studied. On the contrary, *Q*_pyrolysis_ increases as the S/B ratio is increased due to the rise in the water flow rate to be vaporized and heated; i.e., *Q*_pyrolysis_ is the one that contributes the most to *Q*_total_.

Since the pyrolysis and calcination have been identified as the main steps requiring energy input, these energy requirements need to be covered for attaining an energy neutral process.

Next, each of the steps is addressed in the context of heat integration in the PY-SESR option.

#### 3.2.2 Pyrolysis

**Figure 9:**
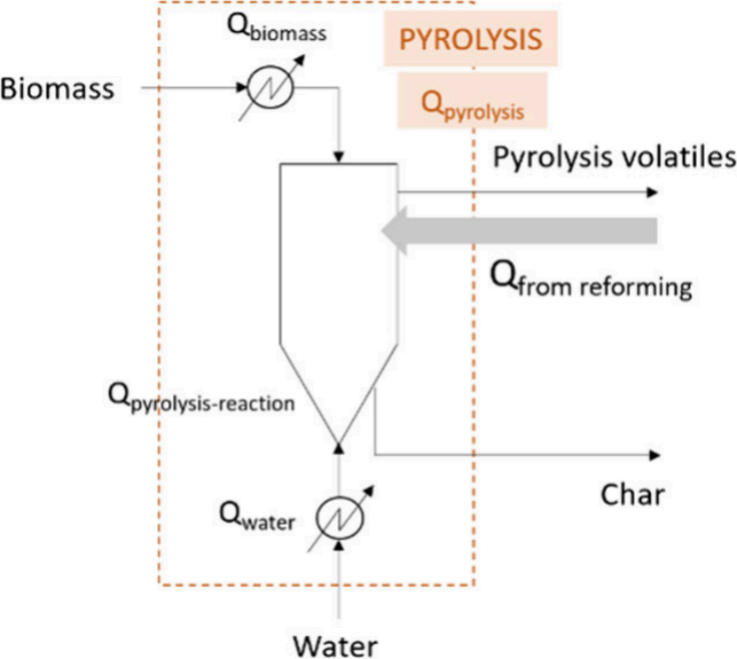
Energy inputs required for the pyrolysis step.

The specific energy inputs required in the pyrolysis step are listed in Figure 9. They are as follows: the energy needed for heating the biomass from 25 to 500 °C (*Q*_biomass_, positive), the energy needed for vaporizing and heating the water from 25 to 500 °C (*Q*_water_, positive), and the energy related to the pyrolysis reaction (*Q*_pyrolysis-reaction_, negative). The net result is positive, so there is a need for energy in this step.

Part of this energy (*Q*_biomass_ + *Q*_pyrolysis-reaction_) is supplied by recovering heat from the SESR reactor (*Q*_from-SESR_), and the remaining (*Q*_water_) is supplied by recovering heat from the reforming products (*Q*_reforming-products_), but also from the SESR reactor (*Q*_water-from-SESR_). According to this scenario, the pyrolysis step is energy balanced.

It is worth noting that although the net energy requirement for the pyrolysis step increases as the S/B ratio is increased, heat integration is hardly affected. This is because the inlet water is heated by recovering heat from the reforming products, which contain unreacted water.

As a consequence, there is flexibility for selecting the desired S/B ratio. In this case, an S/B ratio of 2 was selected for the heat integration configuration. Although one of the advantages of PY-SESR is the possibility of lowering the S/B ratio to 0.5, with H_2_ production and H_2_ purity maintaining at high values, it was decided to use a higher value of S/B ratio for flexibility and practical reasons, such as those related to the reaction rate and catalyst deactivation. Thus, although this study was based on thermodynamic calculations, a higher ratio is recommended to avoid kinetic limitations in real operation. Furthermore, a very low S/B ratio would also make it difficult to attain the desired fluidization regimes in both reactors.

#### 3.2.3 Sorption Enhanced Reforming (SESR)

**Figure 10:**
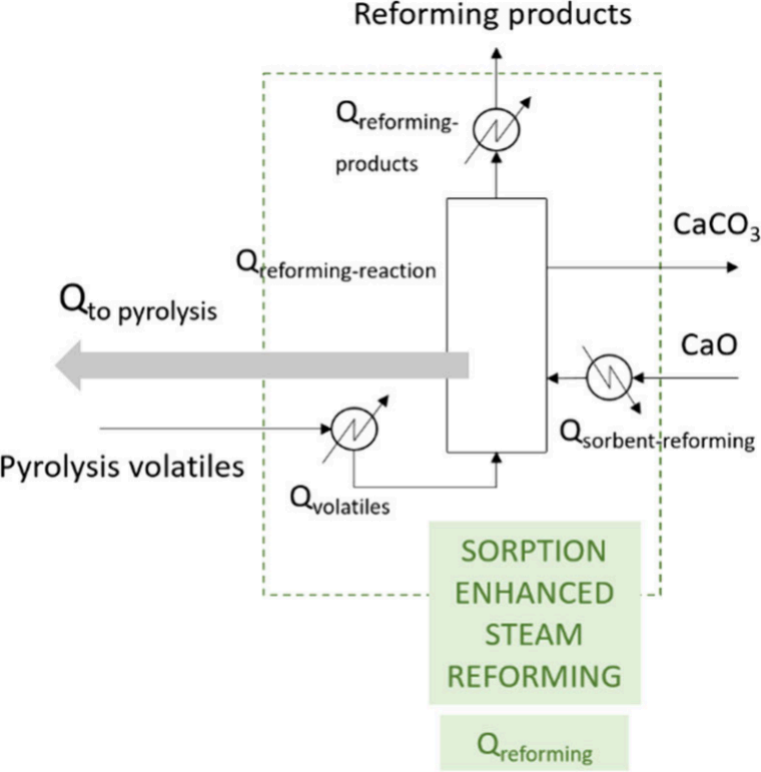
Energy inputs required for the SESR step.

The specific energy inputs in the SESR step are listed in Figure 10. They are the energy needed to heat the pyrolysis volatiles from the pyrolysis temperature (500 °C) to the reforming temperature (600 °C) (*Q*_volatiles_, positive), the energy related to the reforming reaction (*Q*_reforming-reaction_, negative), the energy related to the cooling of the sorbent from the calcination temperature (750 °C) to the reforming one (600 °C) (*Q*_sorbent-reforming_, negative), and the energy related to the cooling of the reforming products to supply heat to the inlet water (*Q*_reforming-products_, negative). The net energy requirement of this step is zero since it is balanced by recovering the excess energy and supplying it to the pyrolysis step. When both the pyrolysis and SESR steps were integrated at the mentioned operating conditions, the temperature of the reformed products was 65.71 °C.

#### 3.2.4 Calcination and Combustion

**Figure 11:**
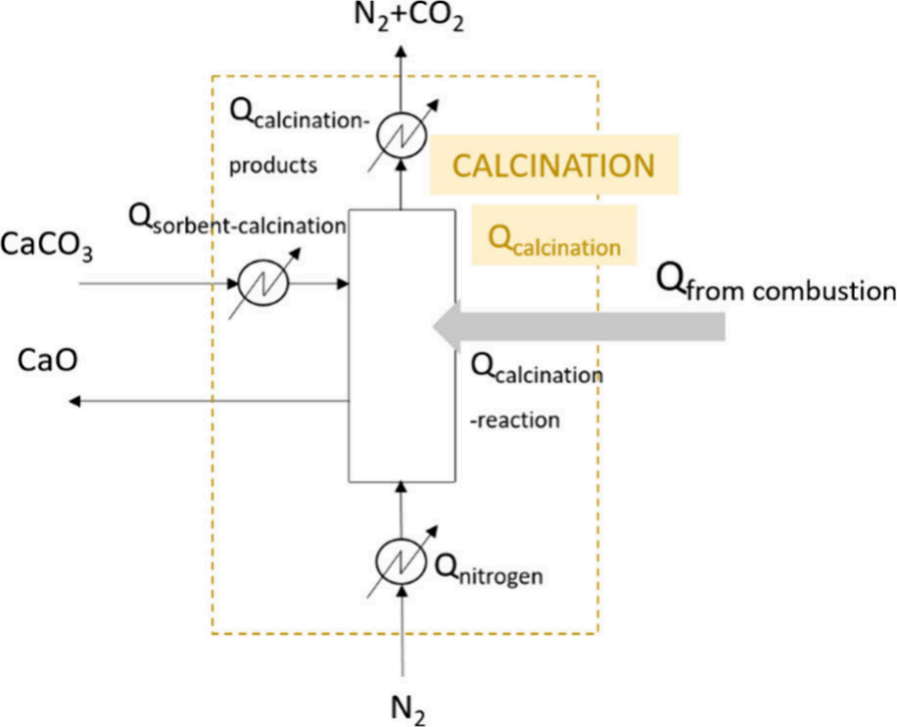
Energy inputs required for the calcination step.

The specific energy inputs for the calcination step are listed in Figure 11. They are the energy needed to heat the sorbent from the reforming temperature (600 °C) to the calcination one (*Q*_sorbent-calcination_, positive), the energy needed to heat the nitrogen feed from 25 °C to the calcination temperature (*Q*_nitrogen_, positive), the energy related to the calcination reaction (*Q*_calcination-reaction_, positive) and the energy related to the cooling of the calcination products from the calcination temperature to the outlet one (depends on the heat removed from this stream) (*Q*_calcination-products_, negative). It is noted that *Q*_sorbent-calcination_ will be supplied once the solids are within the calcination reactor.

**Figure 12:**
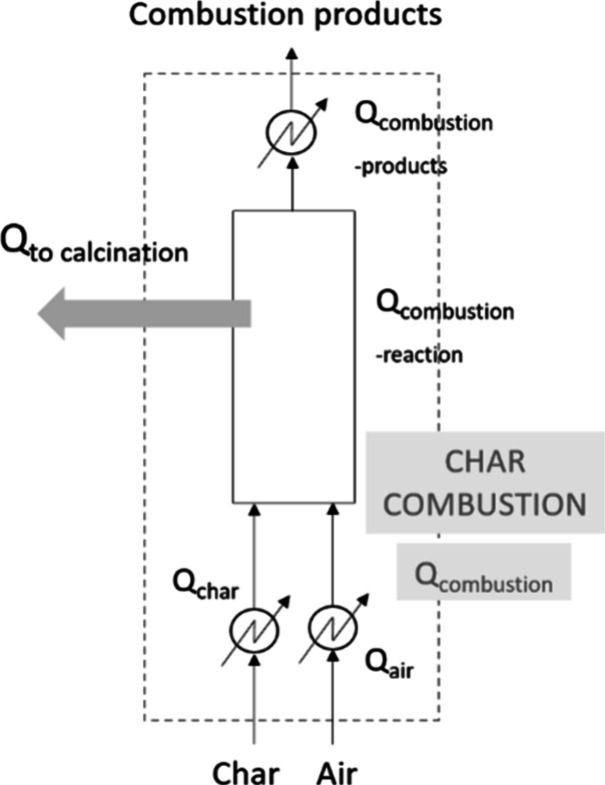
Energy inputs required in the combustion step.

Due to the high endothermicity of the decarbonation reaction, the net energy input needed in this step is considerable. Bearing this in mind, an approach based on supplying heat to the calciner by burning the biochar generated in the pyrolysis step was considered for attaining a thermally autosustained process. The specific energy inputs related to this combustion step are listed in Figure 12.

In particular, the energy related to the char combustion (*Q*_combustion-reaction_) should cover *Q*_calcination-reaction_, *Q*_sorbent-calcination_, the energy required for heating the char from the pyrolysis temperature (500 °C) to the combustion one (*Q*_char_), and the energy required to slightly heat the nitrogen flow for calcination (*Q*_nitrogen-1_) and the air flow for combustion (*Q*_air-1_). The aim of the slight heating of these streams is to ensure flexibility in the heat integration.

The energy for heating the nitrogen for calcination (*Q*_nitrogen-2_) and the air for combustion (*Q*_air-2_) were not considered since they were supplied by the streams of calcination products (*Q*_calcination-products_) and combustion ones (*Q*_combustion-products_), respectively. The energy released by the char combustion (*Q*_combustion-reaction_) was determined based on its heating value.^37^

When the char generated in the pyrolysis at 500 °C is used to cover the calcination and combustion heat requirements, the ceiling calcination temperature reached is 784.25 °C. The heat requirements are those involved in the heating of the char from the pyrolysis temperature to the calcination one (*Q*_char_), the heating of the sorbent from the reforming temperature to the calcination one (*Q*_sorbent-calcination_), and the energy involved in the calcination reaction (*Q*_calcination-reaction_), which decreases slightly as the calcination temperature is increased.

As a remark, there are other biomass types yielding higher amounts of char when subjected to fast pyrolysis at 500 °C.^33^ For instance, citrus waste and rice husk yield 0.293 kg_char_ kg^–1^_biomass_ and 0.252 kg_char_ kg^–1^_biomass_, respectively. They are significantly higher than the value of 0.173 kg_char_ kg^–1^_biomass_ corresponding to that obtained from pine wood sawdust. Thus, the type of biomass has a great impact on the heat integration of the process if the char generated is combusted for energy generation.

It is interesting to note that the calcination temperature is of high relevance due to the following aspects: (i) the decarbonation rate is higher when this temperature is higher,^47^ (ii) high calcination temperatures lead to sorbent microstructural changes due to the sintering, which lead to a lower sorbent capture capacity,^59^ (iii) decarbonation CO_2_ partial pressure is greatly affected. Thus, equilibrium CO_2_ partial pressure at 750 °C is 0.084 bar, whereas it is 1.09 bar at 900 °C (partial pressure determined with the correlation proposed by Ortiz et al.^48^). Therefore, it conditions the concentration of CO_2_ in the gaseous stream required to regenerate the sorbent. Thus, at 900 °C, it would be possible to carry out the sorbent regeneration at atmospheric pressure in a pure CO_2_ atmosphere, which would allow recovering the CO_2_ captured without the need for purification.^60^ A pure CO_2_ product stream could also be obtained at lower temperatures when the operation is carried out under a vacuum.

It should be noted that lower temperatures must be used when the sorbent is calcined together with the catalyst. That is, Ni-based catalysts employed in steam reforming are prone to sintering when exposed for long times at temperatures higher than 700 °C.^61−63^

Taking these aspects into account, a calcination temperature of 750 °C was selected aiming at minimizing the sorbent deactivation and making it possible to regenerate both the catalyst and the sorbent in the same reactor. The operation was carried out at atmospheric pressure. Thus, nitrogen was used as an inert compound to achieve CO_2_ partial pressures lower than those of equilibrium. Specifically, the nitrogen mass flow rate was calculated in order to achieve a CO_2_ partial pressure 30% lower than that of equilibrium. This value could be further varied depending on the fluid dynamics required in the calcination reactor.

As for the combustion temperature, a value of 770 °C was selected, which is 20 °C higher than the calcination temperature, in order to ensure the thermal levels required for heat transfer.

### 3.3 Final Heat Integration Configuration

Based on the previous explanations, the configuration for heat integration in the PY-SESR option is the one shown in Figure 3.

The operation temperatures are given in Table 4.

**Table 4:** Operation Temperatures in the PY-SESR Heat Integration Configuration

stage	temperature (°C)
Pyrolysis	500
SESR	600
Calcination	750
Combustion	770

The mass flow rates of the main streams are listed in Table 5. It is to be noted that the imbalance in the mass balance involving biomass, water, char, and reforming products is a consequence of the CO_2_ capture.

**Table 5:** Mass Flow Rates of the PY-SESR Heat Integration Configuration

stream	mass flow rate (kg h^–1^)
Biomass	100.00
Water	200.00
Char	17.34
CaO	158.90
Gaseous reforming products	15.74
Unreacted water	146.11
Nitrogen	1195.68
Calcination products	1316.49
Air	177.84
Combustion products	195.18

The main results obtained for the process are listed in Table 6. Note that the maximum CaO mass conversion is 78.57%.

**Table 6:** Main Results of the PY-SESR Heat Integration Configuration

H_2_ production (kg_H_2__ kg^–1^_biomass_)	H_2_ purity (mol %)	CaO conversion (mass %)	*Q*_total_ (MJ h^–1^)
0.12	98.59	76.03	–1.76

**Figure 13:**
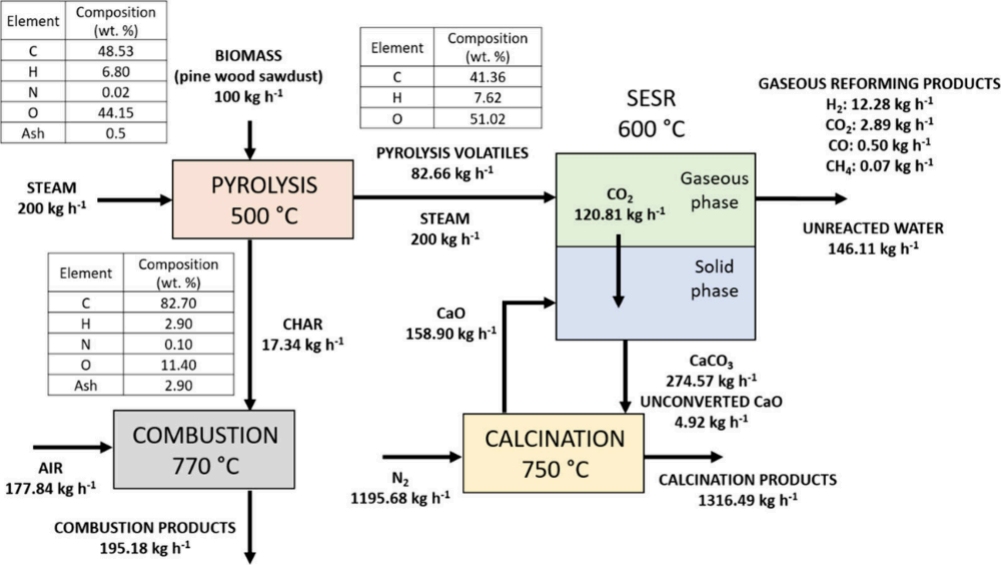
Heat integration configuration mass balance.

A detailed mass balance of the process, based on the considerations mentioned in sections 2.2 and 3.2, is presented in Figure 13.

Finally, the thermal and energy specifications of the process are detailed in Table 7.

**Table 7:** Thermal and Energy Specifications of the PY-SESR Heat Integration Configuration

overall energy term	specific energy term	thermal specification	energy (MJ h^–1^)	total energy (MJ h^–1^)
*Q*_pyrolysis_	*Q*_biomass_	25 to 500 °C	125.62	0.00
	*Q*_pyrolysis-reaction_	500 °C	–25.50	
	*Q*_from-SESR_	-	–100.12	
	*Q*_water_	25 to 500 °C	674.88	
	*Q*_water-from-SESR_	100 to 500 °C	–166.95	
	*Q*_reforming-products_	Water: 25 to 100 °C	–507.93	
		ref. prod.: 600 to 65.71 °C		
*Q*_reforming_	*Q*_reforming-reaction_	600 °C	–305.60	0.00
	*Q*_sorbent-reforming_	750 to 600 °C	–22.64	
	*Q*_volatiles_	500 to 600 °C	61.17	
	*Q*_to-pyrolysis_	-	100.12	
	*Q*_water-to-pyrolysis_	600 °C	166.95	
*Q*_calcination_	*Q*_calcination-reaction_	750 °C	454.07	0.00
	*Q*_sorbent-calcination_	600 to 750 °C	504.16	
	*Q*_nitrogen-1_	25 to 745 °C	941.46	
	*Q*_nitrogen-2_	745 to 750 °C	6.90	
	*Q*_calcination-products_	750 to 98.91 °C	–941.46	
	*Q*_from-combustion_	-	–511.39	
*Q*_combustion_	*Q*_combustion-reaction_	770 °C	–527.14	–1.76
	*Q*_to-calcination_	-	511.39	
	*Q*_char_	500 to 770 °C	10.91	
	*Q*_air-1_	25 to 755 °C	140.00	
	*Q*_air-2_	755 to 770 °C	3.08	
	*Q*_combustion-products_	770 to 137.59 °C	–140.00	

In summary, with the heat integration configuration proposed, it is possible to operate in a thermally autosustained way. However, certain heat exchange rates are tight, and no heat losses were considered. Therefore, a certain amount of biomass, as well as the char, will have to be burnt in the combustor to allow for higher flexibility in a full scale implementation of the process. Another option worth exploring in future studies is oxidative flash pyrolysis. Amutio et al.^64^ determined that 2.7 vol. % of oxygen in the inlet stream is enough for achieving thermally balanced pyrolysis in large scale units.

## 4 Conclusions

The PY-SR and PY-SESR processes were studied based on thermodynamic calculations using the Gibbs free energy minimization method and empirical correlations.

The two processes were compared from the perspective of their energy requirements, H_2_ production and H_2_ purity. PY-SESR was found to be a promising alternative that allows working at lower temperatures and S/B ratios than PY-SR, with H_2_ production and H_2_ purity being higher. Although its energy requirement is higher than that of PY-SR at the same conditions of reforming temperature and S/B ratio, the possibility of operating with lower S/B ratios makes it possible to decrease its energy requirement, with H_2_ production being higher and the product stream almost being pure H_2_. Thus, the energy requirement associated with PY-SR when an S/B ratio of 4 is used is approximately the same as when an S/B ratio of 3 is used in the PY-SESR approach, but H_2_ production and H_2_ purity are improved from 0.118 kg_H_2__ kg^–1^_biomass_ to 0.124 kg_H_2__ kg^–1^_biomass_ and from 67.46 to 98.29 mol %, respectively.

The individual steps in the PY-SESR process were analyzed for a reforming temperature of 600 °C and varying S/B ratios. It was determined that the pyrolysis and the calcination are energy sinks, and the SESR is an energy source. For S/B ratios higher than 0.5, for which there is enough water for the full extension of reforming, the total energy requirement steadily increases due to the great contribution of the energy requirement in the pyrolysis step.

A heat integration configuration for the PY-SESR option is finally proposed. By recovering heat from the product streams, transferring heat from the reforming reactor to the pyrolysis reactor, and burning the char generated in the pyrolysis, it is possible to operate the process in a thermally autosustained way. The study conducted involves certain constraints, such as the nonconsideration of heat losses and use of a calcination temperature of 750 °C and a combustion temperature of 770 °C. However, they may be relaxed by burning some of the biomass along with the char.

Although the results are encouraging, further work is required to assess the influence of certain aspects in real operation at a large scale, as are those related to heat transfer, reaction kinetics, and fluid dynamics.

## Supporting Information Available

It contains the detailed procedure related to the calculations performed in this work (PDF)
