# Supplementary material for: Energy Analysis and Heat Integration in the Joint Process of Biomass Fast Pyrolysis and In Line Sorption Enhanced Steam Reforming
Source: Energy Fuels. 2024 Jul 18;38(15):14402–13. doi: 10.1021/acs.energyfuels.4c02555 (PMC11299529; doi:10.1021/acs.energyfuels.4c02555)
Supplement: Supplementary file 1 — ef4c02555_si_001.pdf [file ef4c02555_si_001.pdf]

Energy analysis and heat integration in the joint process of biomass fast pyrolysis and in line sorption enhanced steam reforming

Pablo Comendador[a], Laura Santamaria[a], Maider Amutio[a], Jon Alvarez[b], Martin Olazar[a]*, Gartzen Lopez[a,c]

[a]Department of Chemical Engineering, University of the Basque Country UPV/EHU, Barrio Sarriena s/n, Leioa, 48940, Spain.

[c]Department of Chemical and Environmental Engineering, University of the Basque Country UPV/EHU, Nieves Cano 12, Vitoria-Gasteiz, 01006, Spain.

[c]IKERBASQUE, Basque Foundation for Science, Plaza Euskadi 5, Bilbao, 48009, Spain.

*Corresponding author: martin.olazar@ehu.es

1. PY-SR alternative

The terms included in the energy analysis in the PY-SR alternative are as follows:

$$Q_{total} = Q_{pyrolysis} + Q_{reforming} \tag{1}$$

$$Q_{pyrolysis} = Q_{biomass} + Q_{water} + Q_{pyrolysis-reaction} \tag{2}$$

$$Q_{reforming} = Q_{volatiles} + Q_{reforming-reaction} \tag{3}$$

They are explained below:

- $Q_{biomass}(MJ\ h^{-1})$: energy requirement for heating the biomass from ambient temperature (25 °C) to the pyrolysis one (500 °C).
- $Q_{water}(MJ\ h^{-1})$: energy requirement for heating the water from 25 °C to the pyrolysis temperature (500 °C).
- $Q_{pyrolysis-reaction}(kJ\ h^{-1})$: energy requirement for the pyrolysis reaction at 500 °C.
- $Q_{volatiles}(MJ\ h^{-1})$: energy requirement for heating the pyrolysis products from the temperature of the pyrolysis (500 °C) to the temperature of the reforming.
- $Q_{reforming-reaction}(MJ\ h^{-1})$: energy requirement needed for the steam reforming step.

The calculations of these terms are detailed below.

1.1. Biomass energy requirement ($Q_{biomass}$)

In order to determine the energy needed for heating the biomass, the next equation was used:

$$Q_{biomass} = \left(C_P^{biomass} \cdot (373 - T_a) + (1 - X_m) \cdot C_P^{biomass} \cdot (T_r - 373) + X_m \cdot \lambda_v\right) \cdot m_{biomass} \tag{4}$$

where $Q_{biomass}$ is the energy needed to heat the biomass from the ambient temperature to the reaction one (MJ h^{-1}), $C_P^{biomass}$ is the biomass specific heat (MJ $kg^{-1}_{biomass}$ K^{-1}), T_a is the ambient temperature (K), X_m is the moisture mass fraction of the biomass (kg_{water} $kg^{-1}_{biomass}$), whose value is 0.1 kg_{water} $kg^{-1}_{biomass}$, T_r is the reaction temperature (K), which was set at 873 K, λ_v is the water latent heat of vaporization at atmospheric pressure (MJ kg^{-1}_{water}), which has a value of 2.26 MJ kg^{-1}_{water} [1] and $m_{biomass}$ is the biomass mass flow rate ($kg_{biomass}$ h^{-1}), which has been set to 100 $kg_{biomass}$ h^{-1}.

The specific heat of the biomass has been determined by using the next equation [2]:

$$C_P^{biomass} = \frac{1.112 + 4.85 \cdot 10^{-3} \cdot (T - 273)}{1000} \quad (5)$$

where $C_P^{biomass}$ is the specific heat of the biomass (MJ $kg^{-1}_{biomass}$ K^{-1}) and T is temperature (K).

1.2. Water energy requirement (Q_{water})

The energy required for heating the water from the ambient temperature to the reaction temperature was determined as follows:

$$Q_{water} = \left(\int_{T_a}^{373} C_P^{water-liquid} \cdot dT + \lambda_v + \int_{373}^{T_r} C_P^{water-vapor} \cdot dT \right) \cdot S/B\, ratio \cdot m_{biomass} \quad (6)$$

where Q_{water} is the energy needed to heat the water from the ambient temperature to the reaction one (MJ h^{-1}), C_P^{water} is the water specific heat (MJ kg^{-1}_{water} K^{-1}), T_a is the ambient temperature (K), T_r is the reaction temperature (K), which was set at 873 K, λ_v is the water latent heat of vaporization at atmospheric pressure (MJ kg^{-1}_{water}), which has a value of 2.26 MJ kg^{-1}_{water} [1], $S/B\, ratio$ is the steam to biomass ratio (kg_{water} $kg^{-1}_{biomass}$) and $m_{biomass}$ is the biomass mass flow rate ($kg_{biomass}$ h^{-1}), which has been set to 100 $kg_{biomass}$ h^{-1}.

The specific heat of the liquid water was determined as follows:

$$C_P^{water-liquid} = \frac{R}{M_w} \cdot (A + B \cdot T + C \cdot T^2) \quad (7)$$

where $C_P^{water-liquid}$ is the specific heat of the liquid water (MJ kg^{-1}_{water} K^{-1}), R is the gas constant (8.314·10^{-3} MJ $kmol^{-1}$ K^{-1})), M_w is the water molar mass (kg $kmol^{-1}$), whose value is 18 kg $kmol^{-1}$, T is temperature (K), and A (8.712), B (1.25·10^{-3}) and C (-0.18·10^{-6}) are constants [1].

The specific heat of the water vapor was determined by using the next equation:

$$C_P^{water-vapor} = \frac{R}{M_w} \cdot (A + B \cdot T + C \cdot T^2 + D \cdot T^{-2}) \quad (8)$$

where $C_P^{water-vapor}$ is the specific heat of the water vapor (MJ kg^{-1}_{water} K^{-1}), R is the gas constant (8.314·10^{-3} MJ $kmol^{-1}$ K^{-1}), M_w is the water molecular weight (kg $kmol^{-1}$), whose value is 18 kg $kmol^{-1}$, T is temperature (K), and A (3.470), B (1.450·10^{-3}), C (0) and D (0.121·10^{5}) are constants [1].

1.3. Pyrolysis heat of reaction ($Q_{pyrolysis-reaction}$)

The pyrolysis heat of reaction (MJ h^{-1}) was determined as follows:

$$Q_{pyrolysis-reaction} = q_{pyrolysis-reaction} \cdot m_{biomass} \quad (9)$$

where $q_{pyrolysis-reaction}$ is the pyrolysis heat of reaction considered, whose value is -0.255 MJ $kg^{-1}_{biomass}$ [2], and $m_{biomass}$ is the biomass mass flow rate ($kg_{biomass}$ h^{-1}), which has been set to 100 $kg_{biomass}$ h^{-1}.

1.4. Heating of biomass pyrolysis volatiles ($Q_{volatiles}$) and reaction heat of the steam reforming ($Q_{reforming}$)

As for the energy input required for heating the pyrolysis volatiles from the pyrolysis temperature (500 °C) to the reforming one (500 °C to 800 °C) ($Q_{volatiles}$) and for the energy input required for the reforming step ($Q_{reforming}$), they were determined by using the simulation Pro II v.2021 software, as mentioned in the main manuscript.

2. PY-SESR alternative

The terms included in the energy analysis in the PY-SESR alternative are as follows:

$$Q_{total} = Q_{pyrolysis} + Q_{reforming} + Q_{calcination} \quad (10)$$

The $Q_{pyrolysis}$ was already defined in equation (2).

The remaining terms are defined as follows:

$$Q_{reforming} = Q_{volatiles} + Q_{reforming-reaction} + Q_{sorbent-reforming} \quad (11)$$

$$Q_{calcination} = Q_{nitrogen} + Q_{calcination-reaction} + Q_{sorbent-calcination} \quad (12)$$

The terms that have not already described are as follows:

- $Q_{sorbent-reforming}(MJ\ h^{-1})$: energy involved in the cooling of the CaO from the calcination temperature to the reforming one.
- $Q_{sorbent-calcination}(MJ\ h^{-1})$: energy involved in the heating of the CaO from the reforming temperature to the calcination one.
- $Q_{nitrogen}(MJ\ h^{-1})$: energy required for heating the nitrogen from the ambient temperature (25 °C) to the calcination one.
- $Q_{calcination-reaction}(MJ\ h^{-1})$: energy required for the calcination reaction.

Next, the calculations are detailed.

2.1. $Q_{sorbent-reforming}$

First, it is important to mention that all the sorbent that comes from the calcination is CaO.

The energy involved in the cooling of the CaO from the calcination temperature to the reforming one was determined as follows:

$$Q_{sorbent-reforming} = \left(\int_{T_{calcination}}^{T_{reforming}} C_P^{CaO} \cdot dT \right) \cdot CaO/B\ ratio \cdot m_{biomass} \quad (13)$$

where $Q_{sorbent-reforming}$ is the energy involved in heating the CaO from the calcination temperature to the reforming one (MJ h^{-1}), C_P^{CaO} is the calcium oxide specific heat (MJ kg^{-1}_{CaO} K^{-1}), the $CaO/B\ ratio$ is the calcium oxide to biomass ratio (kg_{CaO} $kg^{-1}_{biomass}$), which has been set as 1.59 kg_{CaO} $kg^{-1}_{biomass}$ and $m_{biomass}$ is the biomass mass flow rate ($kg_{biomass}$ h^{-1}), which has been set to 100 $kg_{biomass}$ h^{-1}.

The specific heat of the CaO was determined as follows:

$$C_P^{CaO} = \frac{R}{M_{CaO}} \cdot (A + B \cdot T + D \cdot T^{-2}) \quad (14)$$

where C_P^{CaO} is the specific heat of the CaO (MJ kg^{-1}_{CaO} K^{-1})), R is the gas constant ($8.314 \cdot 10^{-3}$ MJ $kmol^{-1}$ K^{-1}), M_{CaO} is the CaO molecular weight (kg $kmol^{-1}$), whose value is 56 kg $kmol^{-1}$, T is temperature (K), and A (6.104), B ($0.443 \cdot 10^{-3}$) and D ($-1.047 \cdot 10^{5}$) are constants [1].

2.2. $Q_{sorbent-calcination}$

First, it is important to mention that not all the sorbent that comes out from the reforming step is $CaCO_3$. Depending on the reforming temperature, the carbonation extent would vary. As consequence, the sorbent that leaves the reformer will be a mixture of CaO and $CaCO_3$.

The energy related to the heating of the CaO from the reforming temperature to the calcination one was determined as follows:

$$q_{CaO} = \int_{T_{reforming}}^{T_{calcination}} C_P^{CaO} \cdot dT \quad (15)$$

where q_{CaO} is the energy related to the change in the CaO temperature from the reforming temperature to the calcination one (MJ kg^{-1}_{CaO}).

The energy related to the heating of the $CaCO_3$ from the reforming temperature to the calcination temperature was determined as follows:

$$q_{CaCO_3} = \int_{T_{reforming}}^{T_{calcination}} C_P^{CaCO_3} \cdot dT \quad (16)$$

where q_{CaCO_3} is the energy related to the change in the $CaCO_3$ temperature from the reforming temperature to the calcination one (MJ $kg^{-1}_{CaCO_3}$) and $C_P^{CaCO_3}$ is the calcium carbonate specific heat (MJ $kg^{-1}_{CaCO_3}$ K^{-1}).

The specific heat of the calcium carbonate was determined as follows:

$$C_P^{CaCO_3} = \frac{R}{M_{CaCO_3}} \cdot (A + B{\cdot}T + D{\cdot}T^{-2}) \quad (17)$$

where $C_P^{CaCO_3}$ is the specific heat of the $CaCO_3$ (MJ $kg^{-1}_{CaCO_3}K^{-1}$), R is the gas constant ($8.314{\cdot}10^{-3}$ MJ $kmol^{-1}$ K^{-1}), M_{CaCO_3} is the $CaCO_3$ molecular weight (kg $kmol^{-1}$), whose value is 100 kg $kmol^{-1}$, T is temperature (K), and A (12.572), B ($2.637{\cdot}10^{-3}$) and D ($-3.120{\cdot}10^{5}$) are constants [1].

Finally, the energy related to the heating of the sorbent from the calcination temperature to the reforming temperature was determined as follows:

$$Q_{sorbent-calcination} = q_{CaO} \cdot m_{CaO} + q_{CaCO_3}{\cdot}m_{CaCO_3} \quad (18)$$

where $Q_{sorbent-calcination}$ is the energy related to the change in the sorbent temperature from the reforming temperature to the calcination one (MJ h^{-1}), q_{CaO} is the energy related to the change in the CaO temperature from the reforming one to the calcination one (MJ kg^{-1}_{CaO}), q_{CaCO_3} is the energy related to the change in the $CaCO_3$ temperature from the reforming one to the calcination one (MJ $kg^{-1}_{CaCO_3}$), m_{CaO} is the calcium oxide mass flow rate from the reforming step to the calcination one (kg_{CaO} h^{-1}) and m_{CaCO_3} is the calcium carbonate mass flow rate from the reforming step to the calcination one (kg_{CaCO_3} h^{-1}).

2.3. $Q_{nitrogen}$

The energy required for heating up the nitrogen from the ambient temperature to the calcination temperature was determined as follows:

$$Q_{nitrogen} = \left(\int_{T_{ambient}}^{T_{calcination}} C_P^{N_2} \cdot dT \right) \cdot m_{N_2} \quad (19)$$

where $q_{nitrogen}$ is the energy needed to heat the nitrogen from the ambient temperature to the calcination one (MJ h^{-1}), $C_P^{N_2}$ is the nitrogen specific heat (MJ $kg^{-1}_{N_2}$ K^{-1}) and), $m_{N_2}$is the nitrogen mass flow rate fed to the calcination step (kg_{N_2} h^{-1}) (determined so that the partial pressure of CO_2 in the calcination step is 30 % lower than the equilibrium value for the decarbonation reaction at the calcination temperature).

The specific heat of the nitrogen was determined by using the next equation.

$$C_P^{N_2} = \frac{R}{M_{N_2}} \cdot (A + B{\cdot}T + C{\cdot}T^2 + D{\cdot}T^{-2}) \quad (20)$$

where $C_P^{N_2}$ is the specific heat of the nitrogen (MJ $kg^{-1}_{N_2}$ K^{-1}), R is the gas constant ($8.314{\cdot}10^{-3}$ MJ $kmol^{-1}$ K^{-1}), $M_{N_2}$is the nitrogen molecular weight (kg $kmol^{-1}$), whose value is 28 kg $kmol^{-1}$, T is temperature (K), and A (3.280), B ($0.593{\cdot}10^{-3}$), C (0) and D ($0.040{\cdot}10^5$) are constants [1].

2.4. $Q_{calcination-reaction}$

The calcination reaction is as follows:

$$CaCO_3 \rightarrow CaO + CO_2 \quad (21)$$

The enthalpy of the calcination reaction has been determined as follows:

$$\Delta H_{calcination}^{T_{calcination}} = \Delta H_1 + \Delta H_{calcination}^0 + \Delta H_2 \quad (22)$$

where $\Delta H_{calcination}^{T_{calcination}}$ is the enthalpy of the calcination reaction (MJ $kmol^{-1}$) at the calcination temperature, ΔH_1 is the enthalpy associated with the cooling of the $CaCO_3$ from the calcination temperature to the standard temperature (298 K) (MJ $kmol^{-1}$), $\Delta H_{calcination}^0$ is the standard enthalpy of the calcination reaction (MJ $kmol^{-1}$), and ΔH_2 is the enthalpy associated with the heating of the reaction products from the standard temperature (298 K) to the calcination one.

ΔH_1 has been determined as follows:

$$\Delta H_1 = \int_{T_{calcination}}^{298} C_P^{CaCO_3} \cdot dT \quad (23)$$

The term $C_P^{CaCO_3}$ has been described above.

$\Delta H_{reaction}^0$ is 0.178 MJ $kmol^{-1}$ [1].

ΔH_2 has been determined as follows:

$$\Delta H_2 = \int_{298}^{T_{calcination}} C_P^{CaO} \cdot dT + \int_{298}^{T_{calcination}} C_P^{CO_2} \cdot dT \quad (24)$$

where $C_P^{CO_2}$ is the specific heat of CO_2 (MJ $kmol^{-1}$ K^{-1}).

The specific heat of CO_2 has been determined as follows:

$$C_P^{CO_2} = R \cdot (A + B{\cdot}T + C{\cdot}T^2 + D{\cdot}T^{-2}) \quad (25)$$

where $C_P^{CO_2}$ is the specific heat of CO_2 (MJ $kmol^{-1}$ K^{-1}), R is the gas constant ($8.314{\cdot}10^{-3}$ MJ $kmol^{-1}$ K^{-1}), T is temperature (K), and A (5.457), B ($1.045{\cdot}10^{-3}$), C (0) and D ($-1.157{\cdot}10^5$) are constants [1].

The energy required for the calcination reaction, at the calcination temperature, was determined as follows:

$$Q_{calcination-reaction} = \left(\frac{\Delta H_{calcination}^{T_{calcination}}}{M_{CaCO_3}} \right) \cdot m_{CaCO_3} \quad (26)$$

where $Q_{calcination-reaction}$ is the energy required for the calcination reaction at the calcination temperature, M_{CaCO_3} is the molecular weight of $CaCO_3$ (kg $kmol^{-1}$), whose value is 100 kg $kmol^{-1}$ and m_{CaCO_3} is the mass flow rate of the $CaCO_3$ to be calcined (kg h^{-1}).

3. PY-SESR heat integration

The process diagram and the overall energy balance for heat integration in the PY-SESR option can be found in the Materials and Methods section in the main manuscript.

Next, the calculations concerning each of the terms of the overall energy balance are addressed.

3.1. Pyrolysis

In the pyrolysis step (operation temperature of 500 °C), the energy balance is as follows:

$$Q_{pyrolysis} = Q_{biomass} + Q_{pyrolysis-reaction} + Q_{from-SESR} + Q_{water} + Q_{water-from-SESR} + Q_{reforming-products} \quad (27)$$

I $Q_{pyrolysis-from-SESR}$ refers to the heat transferred from the SESR step to the pyrolysis step to meet the energy requirement described in equation (28).

$$-Q_{from-SESR} = Q_{biomass} + Q_{pyrolysis-reaction} = 125.62 - 25.5 = 100.12\ MJ\ h^{-1} \quad (28)$$

Furthermore, the term Q_{water} corresponds to the energy requirement related to the heating of the water from 25 °C to 500 °C. The S/B ratio was set to 2, and the biomass mass flow rate was fixed at 100 kg h^{-1}, so the water mass flow rate to heat is 200 kg h^{-1}.

It is to note that the term Q_{water} was balanced by the terms $Q_{water-from-SESR}$ and $Q_{reforming-products}$, as shown in equation (29).

$$-Q_{water} = Q_{water-from-SESR} + Q_{reforming-products} \quad (29)$$

$Q_{water-from-SESR}$ refers to the heat recovery from the SESR reactor and $Q_{reforming-products}$ to the heat recovery from the reforming products.

3.2. SESR

A temperature of 600 °C was considered for the SESR step.

The overall energy balance for the SESR stage is as follows:

$$Q_{reforming} = Q_{reforming-products} + Q_{volatiles} + Q_{reforming-reaction} + Q_{sorbent-reforming} + Q_{to-pyrolysis} + Q_{water-to-pyrolysis} \quad (30)$$

The term $Q_{to-pyrolysis}$ refers to the energy recovered from the SESR reactor to supply the energy needed for the pyrolysis ($Q_{biomass} + Q_{pyrolysis-reaction}$).

The term $Q_{water-to-pyrolysis}$ refers to the energy recovered from the SESR reactor for heating the water fed into the pyrolysis step ($-Q_{water-from-SESR}$) by using all the available energy generated in the SESR step.

The energy related to the cooling of the reforming products ($Q_{reforming-products}$) from the reforming temperature to the temperature needed to cover the heat $Q_{water} + Q_{water-from-SESR}$ has been determined by using the PRO II software.

The energy related to the heating of the pyrolysis volatiles from the pyrolysis temperature to the reforming one ($Q_{volatiles}$) and the energy related to the SESR heat of reaction ($Q_{reforming-reaction}$) have also been determined by using the PRO II software.

3.3. Calcination

As mentioned in the section of Results and Discussion, the calcination temperature selected was 750 °C.

The overall energy balance regarding the calcination step is as follows:

$$Q_{calcination} = Q_{sorbent-calcination} + Q_{calcination-reaction} + Q_{nitrogen-1} + Q_{nitrogen-2} + Q_{calcination-products} + Q_{from-combustion} \tag{31}$$

It is to note that $Q_{nitrogen}$ has been divided into $Q_{nitrogen-1}$ and $Q_{nitrogen-2}$. $Q_{nitrogen-1}$ refers to the heating of the nitrogen from 25 °C to 745 °C, which is a temperature slightly lower than that of the calcination. It is consequence of the fact that water is integrated into the calcination products, whose temperature is 750 °C, and a thermal gradient is required for the heat transfer. $Q_{nitrogen-2}$ refers to the heating of the nitrogen from 745 °C to 750 °C, and was assumed to take place inside the calcination reactor.

The energy related to the cooling of the calcination products ($Q_{calcination-products}$) from the calcination temperature to the temperature needed to meet the demand of $Q_{nitrogen-1}$ has been determined by using the PRO II software.

Taking into account that $Q_{nitrogen-1} = -\, Q_{calcination-products}$, and that $Q_{from-combustion}$ is the heat transferred from the combustion stage to the calcination stage in order to balance the calcination stage ($Q_{calcination} = 0$). The latter has been determined as follows:

$$-Q_{from-combustion} = Q_{sorbent-calcination} + Q_{calcination-reaction} + Q_{nitrogen-2} \tag{32}$$

3.4. Combustion

As mentioned in the main manuscript, the char generated in the pyrolysis has been used as fuel for the combustion stage.

The combustion temperature was set at 770 °C, i.e., 20 °C higher than that of calcination.

It is to mention that the char mass flow rate has been determined based on the char yield (0.1734 $kg_{char}\ kg^{-1}_{biomass}$ [3]) and assuming an inlet mass flow rate of biomass into the process of 100 $kg_{biomass}\ h^{-1}$. The air mass flow rate is the stoichiometric value to burn completely the char, for which the composition determined by Amutio et al. [3] has been assumed

The overall energy balance of the combustion stage would be as follows:

$$Q_{combustion} = Q_{combustion-reaction} + Q_{to-calcination} + Q_{char} + Q_{air-1} + Q_{air-2} + Q_{combustion-products} \tag{33}$$

The term $Q_{to-calcination}$ corresponds to $-Q_{from-combustion}$, which was addressed above.

The term Q_{air}, determined by using the software PRO II, is related to the heating of the air inlet. It has been divided into Q_{air-1} and Q_{air-2}. Q_{air-1} refers to the heating of the air from 25 °C to 755 °C, which is a temperature slightly lower than that of combustion. It is consequence of the fact that air is integrated with the combustion products, whose temperature is 770 °C, so a thermal gradient is required for heat transfer. Q_{air-2} refers to the heating of the air from 755 °C to 770 °C, and was assumed to take place inside of the combustor.

The energy related to the cooling of the combustion products ($Q_{combustion-products}$) from the combustion temperature to the temperature needed to meet the demand of Q_{air-1} has been determined by using the PRO II software. Therefore, $Q_{air-1} = - Q_{combustion-products}$, and so $Q_{air-1} + Q_{combustion-products} = 0$.

As for $Q_{combustion-reaction}$, it was determined as follows:

$$Q_{combustion-reaction} = m_{biomass} \cdot Y_{char} \cdot LHV_{char} \tag{34}$$

where $Q_{combustion-reaction}$ is the energy related to the combustion of the char (MJ h^{-1}), $m_{biomass}$ is the biomass mass flow rate ($kg_{biomass}$ h^{-1}), whose value was set at 100 $kg_{biomass}$ h^{-1}, Y_{char} is the char yield (kg_{char} $kg^{-1}_{biomass}$), whose value is 0.1734 kg_{char} $kg^{-1}_{biomass}$ [3] and LHV_{char} is the lower heating value of the char (MJ kg^{-1}_{char}), whose value is 30.4 MJ kg^{-1}_{char} [3].

It is to note that the same value of $Q_{combustion-reaction}$ has being used independently of the combustion temperature.

As for the Q_{char}, it was determined as follows:

$$Q_{char} = m_{biomass} \cdot q_{char} \cdot Y_{char} \tag{35}$$

where Q_{char} is the energy related to the heating of the char from the pyrolysis temperature to the combustion one (MJ h^{-1}), $m_{biomass}$ is the biomass mass flow rate ($kg_{biomass}$ h^{-1}), whose value was set at 100 $kg_{biomass}$ h^{-1}, q_{char} is the energy needed to heat the char from the pyrolysis temperature to the combustion one per mass of char produced (MJ kg^{-1}_{char}), and Y_{char} is the char yield (kg_{char} $kg^{-1}_{biomass}$), whose value is 0.1734 kg_{char} $kg^{-1}_{biomass}$ [3].

The energy required for heating the char from the pyrolysis temperature to the combustion one, per mass of char produced, was determined as follows:

$$q_{char} = \int_{T_{pyrolysis}}^{T_{combustion}} C_P^{char} \cdot dT \tag{36}$$

where q_{char} is the energy needed to heat the char from the pyrolysis temperature to the combustion one, per mass of char produced (MJ kg^{-1}_{char}), and C_P^{char} is the char specific heat (MJ $kg^{-1}_{char}K^{-1}$).

The specific heat of the char was determined by using the next equation [2]:

$$C_P^{char} = \frac{0.43263 + 0.00209 \cdot T}{1000} \tag{37}$$

where T is temperature (K).

4. References

[1] Smith, J. M.; Van Ness, H. C.; Abbott, M. M.; Swihart, M.T. *Introduction to Chemical Engineering Thermodynamics*; McGraw-Hill, 8th ed., 2018.

[2] Koufopanos, C. A.; Papayannakos, N.; Maschio, G.; Lucchesi, A. Modelling of the pyrolysis of biomass particles. Studies on kinetics, thermal and heat transfer effects. *Can. J. Chem. Eng.* **1991**, 69, 907-915. DOI: 10.1002/cjce.5450690413

[3] Amutio, M.; Lopez, G.; Artetxe, M.; Elordi, G.; Olazar, M.; Bilbao, J. Influence of temperature on biomass pyrolysis in a conical spouted bed reactor. *Resour. Conserv. Recycling.* **2012**, 59, 23-31. DOI: 10.1016/j.resconrec.2011.04.002
